# When do we experience effort?

**DOI:** 10.1007/s11229-025-05387-8

**Published:** 2026-01-06

**Authors:** Eleanor Holton, Richard Holton

**Affiliations:** 1 Princeton Neuroscience Institute, Princeton University, Princeton, USA; 2 Faculty of Philosophy, University of Cambridge, Cambridge, UK

**Keywords:** Effort, Mental effort, Physical effort, Affect

## Abstract

We contend that the experience of effort should be understood as the experience arising from resisting an affective behaviour-guiding signal such as hunger, pain, fatigue, or anxiety. We argue that this provides a more satisfactory account than the cost based accounts that have become popular. We distinguish an account of the experience of effort from an account of effort itself, and argue against the reification of efforts.

Why is it that some actions are experienced as effortful, while others, apparently equally physically or mentally demanding, are not? Why is it indeed that the very same action can sometimes be experienced as effortful, and at other times not? Our aim here is to sketch an account of the experience of effort. Our proposal is this: we experience effort when we resist certain signals, signals that serve to elicit corresponding behaviours. Hunger elicits a behaviour: it prompts us to eat. Pain elicits a behaviour: it prompts us to move, or to inhibit movement, as the case may be. Fatigue elicits a behaviour: it prompts us to stop what we are doing. Anxiety elicits a behaviour: it prompts us to increase our vigilance. And so on. These signals carry direction: they prompt us toward particular behaviours that serve particular purposes. They have distinctive phenomenologies that are strongly valenced; that is what makes them so effective. It is when we resist them, we contend, that we experience effort.

Some behaviour-guiding signals prompt us to perform or inhibit behaviours that are predominantly physical, though they will typically have mental elements. Bodily fatigue primarily leads us to rest physically; it also makes cognitive tasks, say solving a crossword, more effortful. Others prompt us to perform or inhibit behaviours that are predominantly mental, though they, conversely, may have physical elements. Physical performance feels more effortful following mental fatigue (Marcora et al., 2009; Van Cutsem et al., 2017). Correspondingly, the experience of our resistance to these signals might be predominantly physical or mental. We take this to be the basis of the distinction—which we take to be one of degree—between the experience of physical effort, and the experience of mental effort. Since we think that there is similar resistance in both cases, we aim to provide a unified account of these two phenomena.

Some methodological points before proceeding. We aim to give something like a real definition (in the sense of Rosen, 2015) of what it is for an action, mental or physical, to feel effortful. But since both definiens and definiendum remain within the broadly intentional, we do not think that this raises major metaphysical challenges. And let us be clear on what it is that we are trying to define. The term ‘effort’ has been used by many theorists—philosophers, psychologists, neuroscientists—to mean many different things. Our focus is on the *experience* of effort, of what it is for an action to feel effortful. This we take to be a felt property of the action concerned. It is represented in ordinary English when the term ‘effort’ is used as a mass noun: ‘Finishing the course took a great deal of effort’, and so on.^1^ We take such uses to be implicitly referring to an experience—finishing the course involved an effortful experience—even when, as here, there is no explicit reference to one. This is how we shall use the mass noun in what follows. In this sense then, effort just is the experience of effort. It might be objected that this is sloppy: that effort itself should be distinguished from the experience, that the effort is what the experience is an experience *of*. We are rather sceptical of such a claim, and we return in the last section to see what sense can be made of it. For now though, all our talk of effort should be understood in terms of experience. (See Bermúdez & Massin, 2023 for a nice discussion of the issues; in their terms, ours is a resolutely feeling-first account.)

## Some initial cases to motivate the account

We start with a few examples. First, a classic case of mental effort. You have been trying to fix a software problem with your laptop and you are getting nowhere. Your frustration builds, but you keep trying, resisting the urge to turn away. This takes effort. At some point, after yet another failed attempt, the frustration becomes so strong that you shut your laptop screen emphatically. You still feel frustrated— two fruitless hours!—but you no longer feel the effort that you did a minute ago. Here the signal was frustration: it has a distinct phenomenology, and elicits the familiar behavioural response of ceasing the activity (and sometimes that of expressing your frustration rather more destructively). Resisting that signal took effort. Frustration is just one of a class of signals that can arise when performing mental tasks: there are others, phenomenally distinct, such as boredom, or the temptation associated with alternative rewards, that are also effortful to resist.

Now take a case of predominantly physical effort. Imagine you are running along the beach on a beautiful morning after a good night’s sleep. The first few hundred meters hardly feel effortful at all: your legs are light, the sand is soft, everything is wonderful. However, you soon start to get tired and as you do the feeling of effort increases. The signals of oxygen depletion and muscle fatigue begin to dominate. The running becomes effortful when you over-ride these signals and persist. At some point, you stop. Your muscles are still tired and oxygen-depleted, and, you are panting. However, since you have stopped, you are now no longer resisting the signals and so you no longer feel effort. Here, the signals were those of physical fatigue. They bring a distinctive phenomenology, and elicit a familiar behavioural response, namely to stop. Resisting those signals took effort.

Next imagine that you have a powerful headache, but that you are in town, with a list of things still to do, and so you do your best to ignore the pain and continue working through your list. That takes effort. Once you complete your list, you return home, relieved. The headache continues, but as you sit, your head still throbbing, you think: ‘At least I don’t have to do anything else tonight’. The pain continues, but the experience of effort has gone. In that case the behaviour-guiding signal was a distinct type of pain: one with a particular phenomenology that guided you, in this case, to inhibit action. Resisting this signal took effort.

Finally take a case where pain directs you to perform an action rather than to desist from one. Imagine you have a nasty splinter in your finger. A friend offers to remove it with tweezers. ‘Hold still’, they say. As they probe with the tweezers, the pain induces a strong inclination to withdraw your hand, but you resist. That takes effort. The splinter removed, you now withdraw your hand, relieved. It still hurts, but the experience of effort is over. Here effort came, not from resisting a signal to desist from a behaviour, but from resisting a signal to initiate a behaviour—resisting a signal to move your hand away. So here the pain prompted a different behavioural response, but resisting that signal still took effort.

We have described both the experiences of mental effort and of physical effort in the same way. As we said at the outset, we aim to provide a unified account of the two. That is theoretically pleasing, but more fundamentally we think it is a requirement on any successful account, since we doubt that there is any neat distinction between them. Consider again the phenomenology. What should we say of our last two examples, of the headache and the splinter? They clearly involved both physical and mental phenomena. Were they cases of physical or mental effort? And even the first two cases mixed mental and physical factors. As as any athlete will tell you, the effort involved in training and competing is as much mental as physical. Some cases press more in one direction than the other, but even those at one extreme typically involve elements of both.

With the initial motivation for our account in place, we turn to what we take to be the main class of rival views, those that understand effort as a signal of cost, and explain why we think our account does better.

## Cost-based accounts

There has been much research in cognitive neuroscience on how the brain incorporates different costs into decisions (Rudebeck et al., 2006; Wallis & Rushworth, 2014). One popular account of the experience of effort holds that it is involved in this process: it is a signal of the costs associated with continuing with an ongoing activity (Kurzban et al., 2013; Székely & Michael, 2021; Bermúdez & Massin, 2023; Shenhav et al., 2017). So for Kurzban and colleagues the phenomenology of effort corresponds to ‘computations of [the] benefits and costs [of mental tasks] relative to other operations to which the same processes might be applied’, so that subjective effort ‘is the conscious, experienced measurement of the costs – especially the opportunity cost – of continuing the task’ (Kurzban et al., 2013, p. 662).

These costs can be very direct: the signals might indicate the cost of using metabolic resources for instance (physical fatigue), or of damaging one’s body. But they may also indicate something somewhat more abstract: in pursuing one activity, one loses the benefits of pursuing other activities. In other words, as Kurzban and colleagues say, effort could also signal opportunity costs. Since these costs may be either mental or physical, such accounts, like our own, succeed in unifying mental and physical effort (Bermúdez & Massin, 2023).

We think there are two decisive objections to cost-based accounts, independent of issues about future costs. First, there is an ambiguity in how to formalise the idea of costs—whether to conceive of them as relative or absolute—that undermines the apparent unity that any account provides. Second, while many subjective costs influence our decisions, not all of these subjective costs give rise to the experience of effort.

Let us spell out these objections in more detail.

### Absolute versus relative costs

We start with the first worry—that there is an ambiguity in how costs are defined, depending on whether we are talking about *relative* costs or *absolute* costs. Different cases require different approaches. Some examples will make the point. To begin with, take a case that primarily involves a form of mental effort that assumes a *relative* cost. Imagine you have decided to read a difficult book—one which you need to read for your book club—rather than watching television. Let’s say that reading the book has a utility of + 10, and watching television has a utility of + 5. So while reading the book is the best action at the moment, the opportunity cost associated with this action is -5: you are forgoing 5 units of utility that you could achieve. As you start reading the book, it does indeed feel effortful and you are keenly aware of how much easier it would be to turn on the television. However, to your surprise, the book is rather good, and half an hour later it would be hard to put it down. Reading the book no longer feels effortful—quite the opposite. What has happened? The utility of reading the book has gone up, let’s say to + 15. You are completing your book club assignment *and* you’re genuinely enjoying it! Nevertheless, the opportunity costs of this activity haven’t changed—watching television is still equally as good as it was half an hour ago. Yet in this case, the experience of effort has gone. So if the cost theorist is going to explain what is happening, it seems that they will have to say that effort is proportional to the *relative* opportunity costs of missing out on alternative activities (against the benefits of pursuing the current activity) – the position taken by Kurzban and colleagues. If the utility of performing the current action far outweighs any alternatives, the feeling of effort will decrease, tending, if the disparity is large enough, to effortlessness, as in the present case.

Now let’s turn to a case that involves predominantly physical effort. Imagine you are cycling up a hill to your friend’s birthday party. Let’s assume the birthday party—which you know will be fun—has a subjective utility of + 10. But the hill is very steep, and you are panting and your calf muscles are aching as you push on the pedals. Let’s say the subjective cost of this action of cycling up the hill is -5. Since the benefits of cycling up the hill (+ 10) outweigh the costs (-5), it’s worth doing. But it still feels effortful. Crucially, this feeling of effort will be present no matter *how good* this party will be. Suppose half way up the hill you receive a text telling you that, unexpectedly, some good friends will be there. The utility of the party goes up (let’s say it’s now + 15), and you are all the more motivated to make the effort. But you are still acutely aware of the effort you are making to put all your weight into the pedals. Two minutes later you get another call saying that Beyoncé has turned up to the party as a surprise guest – how could it possibly get better? Let’s say the benefit of the party is now + 1000, and at this point if it were a calculation of relative costs to benefits you should expect to feel no effort at all on that hill. But, of course, this does not happen. Although it is possible that adding these additional motivations makes the action feel *less* effortful, (and the excitement might make you less likely to *notice* the effort) there is no way in which cycling up a hill to attend a party can become truly effortless, as in the case of mental cost signals. This suggests that under a cost-based account, effort must be more than the output of a relative cost-benefit calculation—but is unavoidably tied to some absolute costs.

There may be some unusual and interesting exceptions where we do indeed no longer feel physical forms of effort. It is possible that in some cases *other* primitive signals—such as fear—could counteract fatigue. Cycling away from a pursuing lion may feel terrifying but not effortful in the moment. Famously, situations of danger can block pain responses even in the presence of serious injury, and this seems to be an adapted response rather than a malfunction (Heinricher & Fields, 2013). But these are special cases, to be explained by specialised mechanisms associated with particular signals. In general, awareness of the benefits of an action cannot eliminate the experience of physical effort, even if it strengthens our resolve to persist.

This ambiguity about whether we are speaking of absolute costs or relative costs is a problem for a cost-based account of effort, and weakens the proposal that it provides a unifying theory of effort. As the previous examples show, some cases of effort, especially those where physical effort dominates, would belong in the absolute costs category; whereas others, those where mental effort dominates, would be in the relative costs category.

### Identifying the costs that lead to effort

Our second objection to cost-based accounts is that identifying effort as a signal of costs fails to do any real work in identifying effort. All the work, we argue, comes from attempts to identify the *types* of subjective costs that result in feelings of effort. This is because not all costs that influence our decisions are experienced as effortful. Consider two everyday decisions: a child’s decision to stop watching television and do their homework; and conversely their decision to stop doing homework and watch television. Both of these decisions are clearly influenced by costs. The decision to stop watching television is a response to, say, the costs of the teacher’s rebuke if the homework is not done, or the longer term costs of falling behind in the subject. The decision to stop doing the homework is also a response to costs—fatigue and the costs of missing out on a good television episode. So there are costs whichever way the child decides. However it is obvious which of these costs are experienced as effort. It feels effortful to battle with homework, but it very rarely feels effortful to watch television. In other words, resisting some of these costs—such as fatigue—will be experienced as effortful, while resisting other costs—such as the cost of the teacher’s rebuke—will not, despite being key factors in the child’s decisions. So the critical question becomes: for what *types* of cost does resistance lead to an experience of effort? The cost-based account provides no obvious answer.

## Composite views

Might we save the cost-based account by adding further conditions? In effect this is what Bermúdez does, so let us examine his proposal. Bermúdez characterizes the standard cost-based account as follows:

**Unified-Cost**. There is only one feeling of effort for both mental and bodily actions. This feeling represents an action as unattractive, and its unattractiveness level corresponds to the sub-personal estimation of its subjective cost. (Bermúdez, 2023, p. 97)

This idea of a sub-personal estimation of the subjective cost of an action is based on many different factors; it is, he says a ‘global estimation of the action’s subjective costs, as reckoned by a general-purpose expected value calculator’. The feeling of effort is a signal that this cost is negative.

Bermúdez is rightly concerned that this cannot explain why we do not feel effort when we imagine, and are motivated by, future costs. To take his example: the person who imagines cycling up a mountain road can be affected by estimates of its future cost; but it is only if they actually do the ride that they will feel the effort. So effort cannot be simply the estimate of overall subjective cost.

In response Bermúdez moves to what he calls the ‘Single-feeling view’:

**Single-feeling view**. There is only one feeling of effort for both mental and bodily actions. This feeling represents an action as involving the resistance of an impulse to stop the action, and the impulse’s strength corresponds to the action’s estimated subjective cost. (Bermúdez, 2023, p. 99)

Bermúdez here embraces an idea that is much closer to what we are proposing: that effort arises in response to resisting an impulse to stop. Since one can only have a impulse to stop if one is actually performing an action, this avoids the future costs problem. Nevertheless Bermúdez’ Single-feeling view is still a composite account: it maintains the idea that the strength of the impulse is proportional to the estimated subjective cost of the action. But that runs straight into the problems that we raised in the last section: that the idea of cost is ambiguous between the absolute and the relative; and that not all estimated costs give rise to a feeling of effort.

We do not see any useful work that the appeal to costs is doing. Rather than supplementing a cost-based view with further conditions, as Bermúdez does, we argue that we should drop the appeal to costs altogether. It is talk of resisting a signal to stop that is really doing the work in his account, which is what we are proposing. So let us turn to developing that view.

## Characterising behaviour-guiding signals: two unsuccessful approaches

How should we characterise the specific set of signals that are effortful to resist? We start with two characterisations that we think won’t work. To understand the first, suppose we approach the issue from the opposite direction: by looking at which behavioural influences are *not* effortful to resist. Building on Bermúdez’ insight, we might say that it is not effortful to resist behavioural influences concerning things that we anticipate will happen in the *future*. Return to the example of the child doing their homework. In this case, they might be motivated by considerations of what will happen tomorrow, when their teacher asks for the assignment and they shamefully have nothing to hand in. This consideration about future costs motivates their behaviour, but resisting it does not feel effortful.

So perhaps we feel effort when we resist influences that concern *current* behaviour. At first glance this might seem right: it is only effortful to resist the influence of current pain, current hunger, or the opportunity costs of missing out on something good *right now.* It is not effortful to resist the influence of the anticipation of pain, hunger, or missed future opportunity costs, even if these factors might influence the decisions we make. It is effortful to resist muscle fatigue while you are cycling up a mountain, but while the anticipation of this experience of physical effort may influence your decision to stay at the bottom of the hill, the anticipation itself does not itself manifest as the feeling of effort (Massin, 2017; Bermúdez & Massin, 2023).

However, this argument—that the experience of effort concerns behavioural signals related to current outcomes—can only go so far. Not all influences concerning *current* outcomes lead to effortful resistance. Consider someone in the midst of a heated argument with their partner. Despite the fury of the argument, they may be aware of many things: that the argument is pointless; that they are quite possibly in the wrong; that the argument is causing both of them to become very upset; that their time right now would be much better spent doing other things. Yet, although they are resisting these reasons in favour of stopping—reasons related to current bad outcomes—the act of continuing to argue does not feel effortful. On the contrary, it is stopping the argument that takes effort. There are many similar examples: examples where the agent is aware of immediate aversive outcomes that should be influencing behaviour, yet resisting them does not feel effortful.

So here is another try at making the distinction: some influences on behaviour are relatively ‘automatic’ while others are more ‘deliberate’. Perhaps it is resisting the automatic signals which leads to the feeling of effort, while resisting the outputs of deliberation does not. In the previous case, resisting the angry indignation that automatically drives an argument forward may be effortful. While we will still be influenced by the more deliberate reasons to refrain, perhaps resisting their influence on behaviour does not feel effortful. The distinction between automatic and deliberate influences on behaviour has been studied under many guises: ‘System I’ versus ‘System II’ (Kahneman, 2011); ‘habitual’ versus ‘goal-directed’ (Balleine & Dickinson, 1998; Dickinson & Balleine, 2002; Killcross & Coutureau, 2003); ‘model-free’ versus ‘model-based’ (Doya et al., 2002; Daw et al., 2005, 2011). However understood, perhaps effort is felt when we resist these automatic influences—fatigue, pain, fear, anger, and the like—and not when we resist the more deliberate influences (Pessiglione et al., 2025).

This distinction is not quite right either. Consider a canonical example of an automatic influence on behaviour which nonetheless does not lead to effortful resistance: habit. Imagine you are have decided to go to a bar with a colleague after work. At the end of the street, you start to turn left—your usual route home. Your colleague looks confused, and asks, ‘Isn’t the bar this way?’. You realise your error and immediately turn right instead. *Resisting* this automatic behavioural influence —the habitual influence that makes you turn left—does not feel in the least effortful. As soon as the mistake is realised, you immediately change path with no second thought. Contrast this with the earlier cases of pain, fatigue, frustration, temptation—influences which are difficult to resist, even in cases when resisting them is clearly the ‘right thing to do’. Of course there are many cases that are frequently described as habitual that are hard to resist: lighting up a cigarette for instance. But here we think that talk of habit is misplaced, or at least, tells only part of the story. For the ‘habit’ of lighting up a cigarette is motivated by a craving, and that is a behaviour-guiding signal that is certainly effortful to resist.

## Behaviour guiding signals as affective signals?

Since neither of these approaches work, let us stand back and return to the rough characterization of a behaviour guiding signal that we gave at the outset. The signals we are concerned with have (i) a direction which guides the individual to perform, or desist from performing, particular actions, and (ii) a valenced phenomenology.

How should we understand the idea of a valenced phenomenology? First, a clarification. In our discussion so far, we have primarily focussed on behaviour-guiding signals that function through their *negative* valence phenomenology: pain, fatigue, frustration, boredom, hunger, disgust. However, there are many positively-valenced signals which are effortful to resist. A good example is the temptation related to primitive gustatory signals. Videos of young children participating in replications of the marshmallow test (Mischel & Ebbesen, 1970) are familiar: the children must choose between a smaller immediate reward (a marshmallow now) or a larger delayed reward (two marshmallows in fifteen minutes). During this delay period, in which the children sit in front of the marshmallow they are resisting, the effort displayed is tangible—one child picks the marshmallow up to have a mournful sniff before throwing it down and covering their eyes. Here effort is felt in the resistance of a positive behaviour-guiding signal ‘pulling’ towards action—the action of eating the marshmallow. It is not surprising that children experience effort when they resist the immediate appetitive gustatory signals that arise when sitting just a few inches from a marshmallow.

Given that the valence can be positive or negative in these ways, one natural approach would be to think in terms of *affect*. Perhaps the valenced signals we are considering are affective signals. That would align our account with the spirit of the account of emotion that has been proposed in psychology by Frijda (1986, 2007, 2010), and in philosophy, with some modifications, by Scarantino (2014). As Scarantino puts it ‘emotions are action control systems designed to prioritize the pursuit of some goals over others’. We talk of ‘affect’ rather than ‘emotion’ because we want our account to embrace states like pain or hunger that are perhaps not thought of as emotions in ordinary English, but do not see this to be a major revision.

We think that there is much that is attractive about this approach, but, pending future refinement, we think that it has too many problems for us to embrace it outright. For a start, there is the question of whether *all* affective states should be understood as behaviour-guiding signals. As these theorists concede, states like guilt do not obviously point to any particular behavioural response: some people respond to guilt by making amends, others by escaping, still others by becoming angry. We leave open here quite how such states should be treated, but note that there is some plausibility to the thought that, whichever behavioural tendency a particular instance of guilt leads to in a particular individual, there will typically be effort involved in resisting that tendency. This is most obviously the case for someone who represses the anger they feel as a result of their guilt (though admittedly here there is another affect involved); or for someone who faces up to the person they have wronged when they are inclined to escape. But even for people who are strongly inclined to make amends, resisting doing so (perhaps because someone has convinced you that, despite its force, your guilt is in fact unwarranted so that you would be wrong to respond to it) will typically be effortful.

Even if this response is successful, there are difficulties in thinking of the behaviour guiding signals as affective that come from the other direction: from cases of behaviour-guiding signals that don’t obviously seem to involve affect. For a start, consider cases in which the signals are fleeting. Consider the subject engaged in a stroop task; they have a fleeting urge to pick the wrong colour, and that is something that they have to resist. That can be effortful, and we think it fits our overall model, since they are resisting a behavioural signal, but it doesn’t seem to involve much affect. Or consider the subject trying to resist entertaining an unwelcome thought (Wegner et al., 1987); again that can be effortful, and here it is a signal for a mental behaviour that we think is being resisted, but again affect doesn’t seem to be involved.

Another set of cases involve the motivational signals keeping us engaged with goals (O’Reilly, 2020; Molinaro & Collins, 2023; Holton et al., 2024). And since resisting these signals also gives rise to effort, our account had better apply to them. Consider Sam, who is a keen player of video games. She is deeply engaged in a game when her father, despairing of getting her to come down for supper, brings a plateful of food and puts it on the desk beside her. Sam acknowledges the food—she is fully aware that it is there—and yet she continues playing, and the food slowly becomes cold and unappetising. Playing the game is demanding: cognitively, emotionally, perhaps even physically (Bowman, 2018). And yet, captivated by the game, Sam finds it more effortful to stop than to continue; she would need, as we might say, to drag herself away. Sam is captivated by a signal motivating her to finish the game. Resisting that signal would take effort.^2^

What is the signal that prompts Sam to keep playing at such cost? It seems like a force that psychologists would label ‘intrinsic motivation’ (Ryan & Deci, 2000). But perhaps we can say more than this. There are a range of fundamental valenced states that moderate our engagement with goals (Carver & Scheier, 1998, 2013). Anticipation pushes us forward when a goal is approached: consider the marathon runner speeding up as they approach the final lap before the finish line. Frustration pushes us to reconsider when a goal fails: consider the earlier case of an individual struggling with a software problem until frustration triggers goal abandonment.

This framework may explain more perplexing cases of effort, for example some cases of the effort needed to overcome some (though doubtless not all) cases of procrastination. Imagine it is nine o’clock in the morning, and you have set yourself one task for the day: finishing the draft of a manuscript. Yet you spend the first forty-five minutes distracting yourself: checking emails, paying a bill, making another cup of tea. It is with great effort that you eventually get on to the manuscript and commit to working on it, but once you do it becomes completely engaging, and you work through the day with great focus. Many of us recognise the effort that can be needed to settle into a task, even though we know it will be engaging once under way: cases of academic writing like this provide a familiar illustration. O’Reilly suggests this procrastination can be linked to a more primitive behavioural signal around goal engagement (O’Reilly, 2020). As we have seen, once we are absorbed in an engaging goal—Sam, our video game player, is a case in point—it is often hard to break free. We will be, in that sense, committed. But if that is so, there are many potential opportunity costs of being in that state, especially for long goals which are likely to engage us over many hours: there are many other things that we could have been doing, that might themselves bring high reward. So we had better have mechanisms that provide fairly high barriers before we make such substantial commitments. Plausibly then, some cases of procrastination involve a reluctance to embark on an action that is guided by a primitive behavioural signal that is stopping us from committing deeply. And there may be many more of this sort of behavioural signal, with more enigmatic origins and presentation, that will only be identified with more empirical work (O’Reilly, 2020).

With this in mind, we can see how we can handle the earlier example of reading a difficult book, an example that was challenging for a cost-based account. In this case, you struggled at first to start reading the book, but soon became captivated by the story and were no longer tempted to watch television instead. Here we say that the period in which you were deliberating about different goals—the ‘deliberative’ state in the words of Heckhausen & Gollwitzer, 1987—you were influenced by signals pulling you toward alternative options. So at the beginning of settling into a difficult activity of reading you felt the opportunity cost of the alternative option—the temptation of turning on the television—before we commit to the chosen activity in full. However, once you were absorbed in the rewarding activity—once you had entered the implementational state—these signals we replaced by motivational signals provided by pursuit of the goal you had chosen, whether these are from the activity itself, or from the allure of completed tasks—of finishing the chapter or moving to the next level (O’Reilly et al., 2014).

In addition there is recent evidence that engagement with a goal might actually lead to alternative goals being suppressed (Holton et al., 2024): other options become less salient in attention, and the temptation associated with them is dampened. So any signal motivating you to watch the television is reduced and perhaps eliminated entirely. This does not mean that agents are locked into their goals. Importantly, if the chosen course of action fails to deliver the expected rewards—if the agent, in other words, becomes frustrated or bored—then alternative options may start to exert a pull again. Here the agent gets a signal to do something else. All of these signals, those related to goal engagement, to frustration, as well as temptation from attractive alternatives, are all likely to be effortful to overcome. Yet the distinct and characteristic ordering of these signals explains how the same actions can go from feeling extremely effortful (e.g. at the beginning of starting a new activity), to completely absorbing, within moments.

In these cases then, we argue that we are influenced by behaviour guiding signals that do not clearly involve affect, though they do have a distinct phenomenology, even if a fleeting one. What should we say? The obvious move would be to say that not all behaviour guiding signals involve affect. The alternative would be to recast the notion of affect to include these. We leave this open. There has been much debate on whether affect is a natural kind, and on quite how its subjective face fits with the evolved mechanisms that underlie it (Barrett, 2006; Barrett et al., 2025). Many have argued that affect is largely socially determined (Barrett, 2017), and has changed radically over time (Boddice, 2018). Clearly it is a notion that is ripe for future revision. Whether the best revision would allow us to say that all behaviour guiding signals involve this revised understanding of affect, we do not know.

To summarise: we agree with the position taken by many psychologists and effectively summarised by Kurzban and colleagues that ‘the mechanisms that comprise the mind have evolved functions…and that subjective experience can be understood as functioning to motivate adaptive behavior’ (2013, p. 663). Here we have argued there are a set of fundamental behavioural signals—signals that have evolved to guide us towards specific actions and are associated with distinctive affective phenomenologies—that are effortful to resist. This is contrasted with many other influences on behaviour (including more deliberate assessments of costs, as well as habitual influences) that do not give rise to feelings of effort when resisted. Establishing a full taxonomy of affective behaviour-guiding signals which are effortful to resist of course remains an open empirical challenge, but we hope that we have said enough to make it plausible that this is the right path to take.^3^

Finally, while we have already argued extensively that effort should not be characterised in terms of costs, but rather through specific signals, we would like to highlight one other difference between our own view and that of Bérmudez. Bérmudez appeals to the idea that effort accompanies resisting the ‘impulse to stop’. However, as we have seen, effort can be involved in resisting the impulse to continue with an action that we are already performing (the case of the video game), or in resisting the impulse to perform a new action (pulling one’s hand away in the case of the splinter). We need the broader idea of an impulse to behave in a certain way – to stop, to continue, or to withhold an action. As we have argued, these belong to a family of more fundamental behaviour-guiding signals.

## From the property of the experience of effort to the property of effort itself

We finish by returning to an issue raised at the outset. Someone might worry that when we talk of the experience of something, that is not enough; we need to know what it is an experience *of*. We can have experiences of polar bears, but no biologist interested in polar bears would think that we should rest content just talking about the experiences. We want to know what polar bears are in themselves. Might we not think the same of effort: think that we need to know what effort is in itself, what the experience of effort is representing, even if we think of this as a property denoted by a mass noun, and not as an object denoted by a count noun? We are far from convinced that there is a well formed question here, or at least, a question that has an answer; but perhaps there is. In this last section we want to examine how things would stand if there were to be an answer.

Start with the case of pain. We think that pain is primarily a signal: a signal to behave in a certain way. Its phenomenal character is there in order to make that signal effective. Is pain also a representation, a representation of bodily damage or suchlike? Perhaps most philosophers think that it is, and one can see their point: pain is of a particular character, tied to a particular place, with a distinct role to play, so that it can seem representational.

We are not entirely convinced, but we are not going to argue about that here.^4^ Our point is rather that the experience of effort may well be quite unlike pain. We have argued that it is the experience that arises when one resists a behaviour guiding signal. So even if in any particular case the behaviour guiding signal itself should be seen as representational (more plausible in some cases than others), we would need to know a lot more before concluding that the experience that arises from resisting it is also representational. There may be a diverse set of different behaviour guiding signals, which, physiologically, have very little in common; so there may be nothing at the physiological level for the experience of effort to represent. There is a parallel here with the difficulties faced by representational theories of colour. The grounds of a perception of, say, red, are so diverse, involving not just many different reflectant and radiant surfaces, but also sensitive to issues of colour constancy and the like, that it is hard to see it as representing any one property. Of course, one might say that it is representing the colour red, just as we might say that the experience of effort represents the phenomenon of effort, but it is unclear that that has really gained us anything.

Should we at least concede that the experience of effort is a signal? After all, it has a certain phenomenological character. Again we don’t deny that it is *possible* that the experience of effort is itself a signal, in which case the agent would be subject to two sets of signals: the first guiding behaviour and the second indicating whether those first signals were being ignored. On what grounds though should we think of it as a signal? We standardly think of signals as having the function of playing that role, either through design, or in natural cases like this, through evolution. The experience of effort might have evolved to play that role. But equally it might just be an accidental side effect, an evolutionary spandrel that has no good grounds for being treated as a signal.

It is hard to think that we could resolve these questions without knowing more about the neurological mechanisms that give rise to the experience of effort. Here there is certainly nothing like a current scientific consensus. As something like a proof of concept, we take one promising new avenue of research, and say a little about how findings there might bear on the issues of representing and signalling. The approach we will explore is that provided by the idea that the agent may indeed be responding to the depletion of some resource, where this is very broadly construed.

An initial attempt to provide a resource depletion substrate for effort came from proposals that effortful exertions—both mental and physical—depleted glucose (Gailliot & Baumeister, 2007). However, evidence for this proposal has been challenged in recent years (Finley et al., 2019), as indeed has the whole ego depletion framework within which it sat (Vohs et al., 2021). Nevertheless, the idea that the experience of effort arises from the depletion of some resource should not be ruled out—a more recent neuro-metabolic account is more promising. It links the experience of effort to the build-up of potentially toxic substances in the brain outside of the cell—glutamate—that needs to be recycled (Wiehler et al., 2022). This theory proposes that exerting cognitive control, both in overtly mental activity and in overtly physical activity (Blain, 2019), leads to neuronal glutamate release—a process that requires reversal, for example during sleep (Dash et al., 2009). Wiehler and colleagues found that regulation mechanisms in other neural areas respond to this build up by decreasing our willingness to exert effort, which explains why we show cognitive fatigue after a strenuous day of exerting effort.

It remains to be seen whether this recent neuro-metabolic account of effort holds up over time. However, these accounts would suggest that effort might have a unifying description at the biological level. If this turned out to be true, we would predict that metabolic markers of effort exertion would be closely linked to the resistance of behaviour-guiding signals, as characterised in this paper: that is, they may be necessary for it, though perhaps not sufficient (Note, however, that Wiehler and colleagues found that the build up of glutamate correlated strongly with the preparedness to do certain tasks, but not with the feeling of effort itself). And if so, we might think of the experience of effort as representing the depletion of those resources, and perhaps as having evolved a signal of the depletion of these resources. These, though, are big ‘ifs’. Whether or not the experience of effort turns out to be representational, and hence whether there is something that it is the experience of, turns on the answers to these questions. It is not something that can be deduced a priori.

## Conclusion

This paper contains both a positive part, and a negative. The negative, against the cost-based account of effort and its derivatives, stands, we think, quite independently of the positive. Cost-based accounts fail first because they equivocate on the notion of ‘costs’, and second because not all costs influencing behaviour are *felt* as effortful. Identifying *which* influences on behaviour are felt as effortful is precisely the challenge at hand. The positive part identifies experiences of effort as experiences arising when one resists a behaviour guiding signal. So it is a presupposition of our account that the behaviour guiding signals are something on which we can get an independent purchase: we must not simply identify them as those things that generate effort when resisted. This is a substantial commitment inviting future theoretical and empirical investigation.

## Footnotes

1 This is what linguists have called an *intensive* mass noun; see (Van de Velde, 1995; Nicolas, 2002a, b). English (along with many other languages) also contains a count noun sense of ‘effort’. This normally has a very different sense, meaning something like ‘attempt’: ‘She made many efforts to get through the window’. Note that this sentence may be true even if none of her efforts were effortful. We do not aim to analyse this ordinary count noun use; we are not giving an account of attempts. Of course it is open to theorists to introduce a count noun sense of ‘effort’ according to which an effort is an effortful attempt. A number of theorists who use the count noun seem to us to be doing this. We find this linguistically rather confusing (ordinary English does do it, but only in a few lexicalized idioms, normally involving the verb ‘make’: ‘She made a big effort’, etc.); and it also creates difficulties about the individuation of efforts that we think largely spurious. So we stick with the mass noun.

2 Is there a complication here from the fact that game playing is often held to be addictive? We doubt it, but readers concerned about the example can substitute another worthy case which is not generally be held to be addictive: reading, say, or completing an artwork.

3 A further issue that needs some exploration is the relation between the behaviour-*guiding* signals that we are talking about, and those signals (which are typically automatic) that *determine* behaviour: the startle response (Koch, 1999) is a good example. Can these responses can be brought under deliberate control so that they become behaviour-modifying signals that can be resisted with effort? The evidence here is mixed. Attempts to modify the startle response, for instance in pilots, for whom it can be dangerous, typically focus on either avoiding provoking the response in the first place, or on controlling the subsequent effects of it (Woods, 2016).

4 The classic defence of the idea that pain has something like propositional content is given by Armstrong (1968). For a good general discussion of the issues and the problems see (Aydede, 2019). Other theorists have pressed the view that pain experiences do have content, but an imperative content: ‘Move away!’ etc. (Klein, 2015). While we are in agreement with Klein that pain is a signal to action, the correct response in different contexts seems much too variable for there to be a plausible propositional content.
